# Deviation of the Nasal Septum

**Published:** 1902-04

**Authors:** Edgar A. Forsyth

**Affiliations:** Buffalo, N. Y.; Laryngologist, Rhinologist and Otologist to the Emergency Hospital.


### Deviation of the Nasal Septum.

BY EDGAR A. FORSYTH, M. D., Buffalo, N. Y.,
Laryngologist, Rhinologist and Otologist to the Emergency Hospital.

DEFORMITIES of the nasal septum are probably the most frequent of all the exciting causes of catarrhal inflammation in the nasal mucous membrane. A majority of the cases we examine for nasal trouble will show some deformity of the septum. Heyman claims that 90 per cent. of all the cases examined will show deformities, although Morell Mackenzie, in an examination of over 2,000 skulls, found a deviation of the septum in only 75 per cent.

P. Watson Williams says deviation of the septum may almost be regarded as its normal condition, for it is only in comparatively few persons that it is absolutely straight; nearly all nasal septa bulge slightly into one or the other nostril and a deviation becomes pathological only when it obstructs respiration. Zuckerkandl claims deviation does not exist before the seventh year, while others maintain they can be found at any age. Ingals says they may be found in new-born infants, and Jacob has demonstrated by showing anatomical preparations that such deformities exist in the fetus.

There is much difference of opinion in regard to cause, but the majority of observers are inclined to attribute it to injuries.

The symptoms complained of most are nasal obstruction, mouth breathing, dryness of nasal passages and mouth, nasal voice, and post-nasal catarrh. Bosworth says deformity of the septum probably more than any other single cause gives rise to attacks of epistaxis. The stenosis caused by deviation of the septum is a frequent cause of middle-ear disease and affections of the larynx and trachea, and if the deviation presses on the middle turbinated bone we have headache, paroxysmal sneezing with watery discharge, hay fever, asthma and other reflex neuroses. Holbrook Curtis has brought to the notice of the profession that anemia is sometimes due to the nasal obstruction and after relief of the stenosis, improvement took place in his cases to the extent of a regain of oxy-hemoglobin in some instances of over double the former amount.

Treatment of deviation of the nasal septum is necessarily surgical. In some cases where there is a thickening of the septum, the knife or saw will correct the trouble by removing the thickened portion, but in cases where there is a marked deviation without thickening, I believe Asch's operation to be the best.

There are a great many operations for correcting nasal deformity, but Asch's operation has been done a great many times by Asch and others, and has given very satisfactory results. Asch and others doing his operation use general anesthesia, but in my cases I have used cocaine, and in the last one used adrenalin to prevent hemorrhage. I believe there is a great advantage in using local anesthesia, as you can have the patient sit in a chair and you are able to see what you are doing, and in case of hemorrhage you have your patient in an upright position where you can work to better advantage; the blood is not running down the throat, perhaps getting into the larynx. However, since the introduction of supra-renal extract, or better still, adrenalin, nasal operations can be performed with little or no hemorrhage, and the cocaine will deaden the parts so much that they feel very little pain—no more than that caused by sawing off septal spur.

Preliminary operations are sometimes required in cases in which there is a luxation of the triangular cartilage from the columna. Polypi in the concavity should be removed and if they exist on the other side they are probably behind the deviation, and cannot be reached until the deformity is corrected. The middle turbinated bone may be hypertrophied so as to interfere with the patency of the wider nasal fossæ after the septum has been brought back to a vertical plane, or it may interfere with the surgeon's efforts to push the septum back into the vertical position; such cases demand the preliminary removal, either partial or total, of the turbinated bone.

Asch's operation consists in making a crucial incision through the cartilaginous septum, breaking down by the fingers or forceps the bases of the segments thus formed, and the insertion of a hollow splint. This operation is equally effective in any form of deviation. Asch has devised several instruments which he uses to perform his operation, and if his method of operating is followed the result will be satisfactory.

I have operated five times on marked deviation of the nasal septum and have had good results in all but one case; that was my first. The patient was very timid and fainted while his nose was being examined. However, I managed to do operation and, as I had no hollow splints, used a tampon made of gauze and gave him instructions to come and see me the next day. The gauze came out the next day and he did not come to see me until the third day, and then he would not allow me to put another tampon in the nose. The result was that deviation resumed its former position. This was my only failure, and that was due entirely to the timidity of the patient. The other cases were operated upon in the same manner but instead of a tampon of gauze I used Mayer's modification of Asch's splint, which was removed every day and cleaned, and nose sprayed out and splint put back again. After the first week the patient can take the splint out daily and clean it and report to physician every two or three days for inspection until splint can be removed, which will be at the end of four or five weeks. Inserting the splint at first is quite painful in some cases; if so, it will be necessary to use cocaine spray before inserting it.

I have had no perforation of the septum, although it does sometimes follow the operation. Asch says perforation following the operation is so rare as not to be considered as a probable sequence and the few cases he has seen were due to cachexia and to unskilful performance of the operation.

326 FRANKLIN STREET.

